# Vagal Tank Theory: The Three Rs of Cardiac Vagal Control Functioning – Resting, Reactivity, and Recovery

**DOI:** 10.3389/fnins.2018.00458

**Published:** 2018-07-10

**Authors:** Sylvain Laborde, Emma Mosley, Alina Mertgen

**Affiliations:** ^1^German Sport University Cologne, Cologne, Germany; ^2^EA 4260 Normandie Université, Caen, France; ^3^Southampton Solent University, Southampton, United Kingdom; ^4^Bournemouth University, Bournemouth, United Kingdom; ^5^University of Luxembourg, Luxembourg, Luxembourg

**Keywords:** heart rate variability, vagal tone, parasympathetic activity, RMSSD, RSA, HF, self-control, executive functions

## Abstract

The aim of this paper is to set the stage for the vagal tank theory, showcasing a functional resource account for self-regulation. The vagal tank theory, building on neurophysiological, cognitive and social psychology approaches, will introduce a physiological indicator for self-regulation that has mainly been ignored from cognitive and social psychology, cardiac vagal control (also referred to as cardiac vagal activity). Cardiac vagal control reflects the contribution of the vagus nerve, the main nerve of the parasympathetic nervous system, to cardiac regulation. We propose cardiac vagal control to be an indicator of how efficiently self-regulatory resources are mobilized and used. Three systematic levels of cardiac vagal control analysis are suggested: resting, reactivity, and recovery. Based on this physiological indicator we derive the metaphor of the vagal tank, which can get depleted and replenished. Overall, the vagal tank theory will enable to integrate previous findings from different disciplines and to stimulate new research questions, predictions, and designs regarding self-regulation.

## Introduction

How healthy are individuals? How effective is their thinking, their stress management, their emotion regulation? How effective are they at developing social relationships? Wholly, how efficient are their self-regulation mechanisms driving their behavior? Surely, you think that the precise answer to each of these questions would take hours and the use of dozens of measures such as cognitive tests, questionnaires, blood analyses, electrocardiogram measurement, electroencephalogram measurement, and so on. This would be completely right. But let’s now imagine that there would be a simple way to summarize all this information in one indicator. The aim of this paper is to introduce the vagal tank theory, a physiological metaphor based on what we propose to be such summary indicator, cardiac vagal control. Building on neurophysiological, cognitive, and social psychology accounts, the vagal tank theory will enable to derive specific research questions, predictions, and research designs that will serve an interdisciplinary understanding of self-regulation.

## Cardiac Vagal Control: A Physiological Indicator of Self-Regulation

The metaphor of the vagal tank is based on the functioning of the parasympathetic nervous system, and more specifically the functioning of its main nerve, the vagus nerve, and its contribution to cardiac functioning, which we coin here cardiac vagal control (Thayer and Lane, 2000; Thayer et al., 2009; Chapleau and Sabharwal, 2011; Smith et al., 2017). Cardiac vagal control is also referred to sometimes as vagal tone, vagal activity, or parasympathetic activity; however, those terms do not refer unambiguously to the contribution of the vagus nerve to cardiac functioning, the term “cardiac” being necessary here to depict the phenomena of interest. Cardiac vagal control can be assumed to index the ability of the vagus nerve to alter heart rate with high responsivity, precision, and sensitivity. It seems largely accepted that the heart influences behavior and this relationship is reciprocal (Sgoifo et al., 2009). For more than 150 years, which included the seminal work of the French physiologist Claude Bernard (Thayer and Lane, 2009), the connection between the heart and the brain through the vagus nerve has received the attention from researchers to understand its influence on self-regulation. Self-regulation refers here to the psychophysiological processes that enable goal-directed behavior over time and across changing circumstances, as well as to the processes that maintain health in an organism (Karoly, 1993; Thayer et al., 2009).

Two main theoretical accounts explain the links between cardiac vagal control and self-regulation: the polyvagal theory (Porges, 2007b) and the neurovisceral integration model (Thayer et al., 2009; Smith et al., 2017). The polyvagal theory (Porges, 2007b) specifies that cardiac vagal control facilitates prosocial behavior through appropriate physiological and behavioral states. The neurovisceral integration model (Thayer et al., 2009) postulates that cardiac vagal control is associated positively to a large range of positive outcomes regarding executive functions, emotion, and health, displaying overall a better self-regulation of the organism (Thayer et al., 2009, 2012). The vagal tank theory will primarily rely on the neurovisceral integration model, given its precise description of self-regulation at the cognitive level, specific to executive functions, which will later help to bridge the gap with cognitive and social psychology. From a neuroanatomical perspective, the brain structures involved in self-regulation and those involved in cardiac control largely overlap, and specifically regarding the prefrontal cortex (Thayer et al., 2009, 2012; Beissner et al., 2013). Functionally, the links between cardiac vagal control and self-regulation can be explained by a functional network linking the heart to the prefrontal cortex (Thayer et al., 2009, 2012), and through the physiology underlying the functioning of the vagus nerve.

### Neurophysiological Underpinnings of the Vagal Tank

There are 12 cranial nerves, the tenth of which is the vagus nerve. The vagus nerve is the most important nerve of the parasympathetic nervous system (Porges, 2007a; Brodal, 2010). It is composed of 80% afferent sensory fibers (sending signals from the body to the brain) and 20% efferent motor fibers (carrying information from the brain to the body). All branches of the vagus nerve with visceral efferent fibers also contain afferent sensory fibers, which makes it a highly sensitive nerve (Howland, 2014). As its name implied (the Latin translation of vagus means *wandering*), the vagus nerve branches to widespread regions of the body (Brodal, 2010), its fibers innervating most organs in the body including the gastrointestinal and cardiovascular systems (Chang et al., 2003; Brodal, 2010). Vagal fibers release acetylcholine as neurotransmitter (Brodal, 2010). To sum up, because of its extensive network the vagus nerve allows for wide spread fast acting communications within the body.

Regarding vagal efferent fibers, those that stimulate motor action, we are particularly interested here in those innervating the heart and modulating its intrinsic activity through the sinus node, which determines heart rate, as we describe later. Importantly, from the two branches of the autonomous nervous system, the sympathetic and parasympathetic, the sympathetic influence on the heart is too slow to produce beat-to-beat changes (Jose and Collison, 1970), and the heart will be mainly under parasympathetic inhibitory influence through vagal efferent fibers (Jose and Collison, 1970; Saul, 1990). This cardiac autonomic balance is a way for the organism to favor energy conservation.

Regarding vagal afferent fibers, those that are linked to sensory actions, they are largely scattered through key organs in the human body. This gives the vagal afferent system an important adaptation role as a detector of immune-related events in the human body. This peripheral sense allows for an internal signal that can generate the appropriate autonomic, endocrine, and behavioral responses via central reflex pathways going through the nucleus of the solitary tract (Berthoud and Neuhuber, 2000). Vagal afferent fibers also contribute to the perception of pain, and thus can be considered as effective pain mediators via central reflex pathways (Berthoud and Neuhuber, 2000). These internal inputs are then integrated to external inputs, which helps to shape the appropriate response. Overall, we can say that the vagus nerve plays an important role in the integration of interoceptive information and in organizing a response with appropriate adaptive modulatory feedback (Yuan and Silberstein, 2015).

A functional network based on brain structures is suggested to facilitate the organization and regulation of vagal afferent and efferent activity (Berthoud and Neuhuber, 2000). The idea that cardiac vagal control is the phasic output of a central processing system which integrates sensory inputs from a variety of afferent sources is widely accepted (Fallen et al., 2001). More specifically, it is expected that the central nervous system is supporting goal-directed behavior, adaptability, and hence self-regulation, based on a functional unit called the central autonomic network (Benarroch, 1993), on which the neurovisceral integration model is based (Thayer et al., 2009). Structurally, this central autonomic network includes different brain structures under the organization of the prefrontal cortex (for details, see Benarroch, 1993; Thayer et al., 2009). This network regulates information flowing bidirectionally between lower and higher levels of the central nervous system. The primary output of the central autonomic network is the sympathetic and parasympathetic activity sent to the heart via the stellate ganglia and vagus nerve. The active interplay of these nerves result in an output in the sinus node provoking the time variability observed between each heart beat, referred to as heart rate variability (HRV), which is mainly under the influence of cardiac vagal control (Levy, 1990). Moreover, afferent (sensory) information from the periphery (end organs that are fed by the circulatory system such as the heart and those contributing to the immune system) are fed back mainly through the vagus nerve (Berthoud and Neuhuber, 2000). This then links back to the central autonomic network, which makes up its output (vagal efferent activity) as an indicator of central-peripheral neural feedback and central nervous system-autonomous nervous system integration (Benarroch, 1993; Berthoud and Neuhuber, 2000; Thayer et al., 2009). Therefore the neurophysiological underpinnings of cardiac vagal control demonstrate the wide reaching influences it has over the body and how this may feed into self-regulatory behaviors.

### Role of the Prefrontal Cortex: Person/Environment Integration to Enable Goal-Directed Behaviors

As we mentioned in the previous section, the prefrontal cortex plays an important role in the organization of the central autonomic network responsible for cardiac vagal control. Similarly, it is important to understand how the prefrontal cortex regulates information from internal sources and from the external sources to adapt behavior. The functions of the prefrontal cortex regarding this aspect can be understood within the biological framework of the perception-action cycle (Fuster, 2015). The perception-action cycle represents the flow of information processing between the organism and its environment in a sequence of goal-directed actions. Meaning that our behavior is driven by our goals and consequently this shapes how we select and process information in our environment. Simple and automatic behaviors will engage only the lower levels, where the cycle will run through the spinal cord and subcortical structures. On the contrary, goal-directed behaviors engage the neocortex and the connections between prefrontal and posterior association cortex. The prefrontal cortex is thus located at the summit of the perception-action cycle, integrating across time sensory internal and external information with actions towards a goal (Fuster, 2015). Combining the central autonomic network and the perception-cycle approach illustrates the central role of the prefrontal cortex in goal-directed behavior and hence self-regulation mechanisms. This level of functioning allows the prefrontal cortex to play a specific role in influencing the self-regulatory mechanisms depicted by the vagal tank metaphor we introduce in the next section.

### Vagal Tank Metaphor: Linking Neurophysiology to Cognitive and Social Psychology

So far we have considered self-regulation from a purely neurophysiological perspective (Thayer et al., 2009). Given the range of phenomena covered linked to cardiac vagal control, it is important for further theoretical development to broaden our horizons and discuss how self-regulation has been approached by other scientific disciplines. Another key area for self-regulation has been established in cognitive and social psychology (Baumeister et al., 2007; Hagger et al., 2010; Kurzban et al., 2013; Inzlicht et al., 2014; Kotabe and Hofmann, 2015). We note here that those disciplines have often referred to self-regulation in terms of self-control, but for a matter of clarity, unless referring to the integrative theory of self-control (Kotabe and Hofmann, 2015), we will use the term self-regulation in this paper. self-regulation actually encompasses self-control: self-control referring to the deliberate, conscious, effortful substrate of self-regulation, while self-regulation itself includes all aspects of an organism’s regulation, such as homeostatic processes like maintaining a constant body temperature (Baumeister et al., 2007). Hence, self-regulation is here more suited for the vagal tank theory because of its inclusion of neurovisceral processes (Thayer et al., 2009).

#### Previous Theories Surrounding Self-Regulation From A Social and Cognitive Psychology Perspective

Starting with social psychology, the strength model of self-control (Baumeister et al., 2007) has been developed to explain findings coined with the term ego depletion. Ego depletion refers to the fact that self-control appears vulnerable to deterioration over time from repeated exertions, like a muscle getting tired. It seems then that self-regulation depends on a limited resource that becomes depleted by any self-control tasks, causing performance decrements in any other tasks involving self-control (Baumeister et al., 2007). Subsequently, researchers have endeavored to find a physiological resource underpinning self-regulation, and at the same time debated on the evolutionary purpose of such resource-based functioning (Baumeister et al., 2007; Hagger et al., 2010; Kurzban et al., 2013; Inzlicht et al., 2014). One physiological resource that gave initial promise was glucose (Gailliot et al., 2007); however, it failed to resist a more scrutinized examination (for detailed arguments, see Inzlicht et al., 2014). Moreover, the existence of the ego-depletion effect itself has been recently seriously challenged by a registered replication report based on a large sample size (Hagger et al., 2015), increasing the need to understand the physiological underpinnings of self-regulation processes.

The current views on self-regulation from a cognitive perspective argue differently regarding the existence of a physiological resource: on the one hand, some cognitive accounts assume that self-regulation is essentially driven by motivation (Kurzban et al., 2013; Inzlicht et al., 2014, 2015). For example, the process model argues that self-regulation is driven by a switch between labor and leisure goals (Inzlicht et al., 2014). Overall, those motivational accounts discard the existence of a resource (Kurzban et al., 2013; Inzlicht et al., 2014, 2015). On the other hand, the recent integrative theory of self-control (Kotabe and Hofmann, 2015) does not discard the idea of a resource, and argues for an interaction between control motivation and control capacity. Control capacity is here thought to be sustained by a resource, and Kotabe and Hofmann (2015) points toward a cognitive one, directed attention (Kaplan and Berman, 2010). Furthermore, evidence based on studies involving physical fatigue would question the fact that self-regulatory fatigue would be due only to motivational elements, and argue instead for the contribution of physiological components in this process (Evans et al., 2015). The role of the vagus nerve is particularly underlined, given the widespread distribution of its afferent fibers within the body, which makes it a good candidate to transmit information related to a number of important aspects of the body, such as nutrient availability, infections, and cardiorespiratory states. We will build on this view from the integrative theory of self-control (Kotabe and Hofmann, 2015) and on the evidence based on physical fatigue (Evans et al., 2015) for our vagal tank theory, to assume that control motivation interacts with control capacity as indexed by cardiac vagal control to predict self-regulation behavior.

Beyond their opposing view on the existence of a resource, what is striking is that none of the leading theoretical accounts on self-regulation from a cognitive perspective (Kurzban et al., 2013; Inzlicht et al., 2014, 2015; Kotabe and Hofmann, 2015; Friese et al., 2018) mention cardiac vagal control as a potential underlying physiological resource, and do not even refer to the initial link established by Segerstrom and Nes (2007). Segerstrom and Nes (2007) used a classical ego depletion paradigm (i.e., a paradigm used in social psychology to investigate self-regulation failure) to establish that cardiac vagal control may reflect self-regulatory strength, effort, and fatigue, hence pointing towards its potential role as a resource. This initial endeavor to investigate the role of cardiac vagal control as a physiological resource underlying self-regulation has received very little follow-up so far in cognitive and social psychology (for an exception, see Reynard et al., 2011). At this point, we clarify our viewpoint on whether cardiac vagal control should be considered as a physiological resource. Within the vagal tank theory cardiac vagal control is not to be seen as a resource itself, but should be considered as a physiological indicator reflecting how efficiently self-regulatory resources are mobilized and used, as we detail below.

Despite the lack of further empirical scrutiny in cognitive and social psychology, the fact that cardiac vagal control may be a physiological indicator that can reflect the use of self-regulation resources would be theoretically supported. It is important to note that the existence of the hypothesized resource by social psychologists has been mainly evidenced with executive functions (Baumeister, 2002). Executive functions are high-level cognitive functions that serve goal-directed behavior, which are essentially supported by prefrontal functioning, and are assumed to be a prerequisite for self-regulation (Barkley, 2001; Hofmann et al., 2012). This would match the neurovisceral integration model, as it postulates a specific link of cardiac vagal control with executive functions but not with non-executive functions (Thayer et al., 2009). Moreover, if we consider the seven areas for self-control identified by Baumeister et al. (2007) – control of thoughts, control of emotions, control of attention, control of impulses, cognitive performance, choice and volition, and social processing – one element is striking: all those areas could be related to phenomena indexed by cardiac vagal control (Porges, 2007b; Thayer et al., 2009, 2012). In this context, proposing cardiac vagal control as a physiological indicator indexing resources underlying self-regulation for cognitive and social psychology, based on Segerstrom and Nes (2007), would be a valid assumption. The added value of such consideration would be to enable further theoretical enhancements of the neurovisceral integration model, considering the integration to the cognitive and social psychology literature.

We should report that a recent meta-analysis linking HRV (from which are calculated, among many others, the indicators reflecting cardiac vagal control) and self-control, considered here in the sense of basic cognitive processes, did not support a clear link between both (Zahn et al., 2016a). However, this meta-analysis suffered from several drawbacks at the theoretical and methodological levels, as presented by Laborde and Mosley (2016) – for a response, see Zahn et al. (2016b). First, it was not based on a specific theoretical framework, and focused on heart rate variability instead of cardiac vagal control, which may have lead to a non-exhaustive selection of studies. Second, it considered only resting heart rate variability and not its reactivity, while reactivity plays an important role in adaptation (Beauchaine et al., 2007; Porges, 2007a). Finally, the meta-analysis did not make clear that self-regulation should be assessed concomitantly with heart rate variability assessments. In fact, even if cardiac vagal control is considered as rather stable (Bertsch et al., 2012), there are many situational influences on heart rate variability measurements that may decrease the links with self-regulation in case they are not assessed during the same session (e.g., van Eekelen et al., 2004).

#### Summary: The Vagal Tank Theory

To summarize, we build our vagal tank theory on two sets of literature: on the one hand, based on the evidence from neurophysiology regarding the role of cardiac vagal control in reflecting self-regulation mechanisms (Thayer et al., 2009), and on the other hand, based on the arguments for a resource underlying self-regulation performance from a cognitive and social psychology perspective (Baumeister et al., 2007; Hagger et al., 2010; Kotabe and Hofmann, 2015). Following initial empirical work combining those two main theoretical perspectives, with Segerstrom and Nes (2007) who proposed cardiac vagal control to be a resource reflecting self-regulatory strength that can be depleted and replenished, we introduce the physiological metaphor of the vagal tank. The vagal tank provides a metaphorical basis to describe the functioning of cardiac vagal control, which depicts adaptive physiological functioning. More specifically, the vagal efferent fibers between the central autonomic network and the sino-atrial node represent the vagal tank, with cardiac vagal control acting as an indicator reflecting how effectively resources underlying self-regulation are mobilized and used (based on Benarroch, 1993; Berthoud and Neuhuber, 2000; Thayer et al., 2009). Cardiac vagal control (i.e., vagal efferent activity to the heart) is the output of the central autonomic network, and therefore serves as an indicator of central-peripheral neural feedback and central nervous system-autonomous nervous system integration, justifying its role as a general summarizing self-regulation index. In other words, we do not consider cardiac vagal control being a resource itself. We rather assume it has a barometer role, and that different levels and change patterns (i.e., direction and magnitude) of cardiac vagal control in the vagal tank can demonstrate adaptive or maladaptive self-regulation according to the situation and task at hand, and thus be an indicator of the self-regulation mechanisms underlying human behavior. In the next sections we present how cardiac vagal control can be measured as well as the time points to consider, on which we build the predictions of the vagal tank theory, aiming to illustrate how the vagal tank underlies human behavior.

### Cardiac Vagal Control: How to Identify It?

**FIGURE 1:**
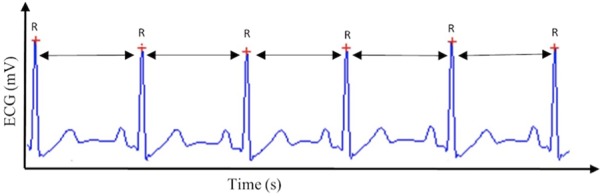
Heart rate variability. This figure displays the method by which heart rate variability, and subsequently cardiac vagal control, is calculated, based on the R–R intervals extracted from the electrocardiogram (ECG) signal.

Cardiac vagal control can be tracked efficiently through a non-invasive, cost-effective marker: HRV (Chapleau and Sabharwal, 2011). HRV is the variability in time between successive heartbeats (see **Figure 1**) and demonstrates the interplay between the sympathetic nervous system and the parasympathetic nervous system (Appelhans and Luecken, 2006). Cardiac vagal control is reflected in several HRV parameters (Malik, 1996): for the time-domain, the root mean square of the successive differences between adjacent normal RR intervals (RMSSD), the percentage of successive normal RR intervals differing more than 50 ms (pNN50), and the peak-valley analysis (Grossman et al., 1990), a time-domain filter dynamically centered at the exact ongoing respiratory frequency (Grossman et al., 1990). For the frequency-domain, high-frequency (HF) reflects cardiac vagal control, but only in case breathing frequency is comprised between 9 and 24 cycles per minute (Malik, 1996).

**FIGURE 2:**
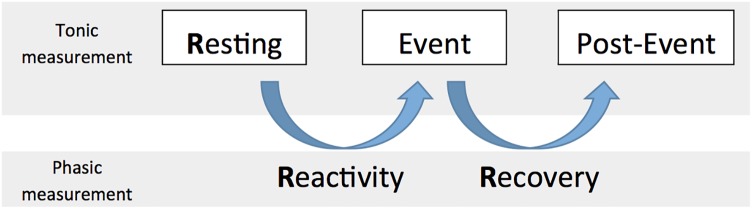
The 3 Rs of cardiac vagal control: Resting, Reactivity, and Recovery.

When specifically focusing on cardiac vagal control, it is important to take into account the circumstances we are measuring cardiac vagal control in (Laborde et al., 2017b). In this paper, we consider tonic cardiac vagal control as the value at a specific time point measurement, and phasic cardiac vagal control as the change of values between two time point measurements. Both of these properties of cardiac vagal control, either tonic or phasic, are important to consider regarding adaptation abilities of the organism (Porges, 2007a; Thayer et al., 2012). In metaphorical terms, tonic refers to the points at which the tank is measured to see how well self-regulatory resources can be used in order to foster adaptability, while phasic refers to the changes in the levels of the vagal tank, which may determine how well the individuals adapt to the situation. We describe below (see also **Figure 2**) the tonic and phasic properties of cardiac vagal control according to three aspects: resting (tonic), reactivity (phasic), and recovery (phasic), which will be important to specify the predictions of the vagal tank theory.

### The Three Rs of Cardiac Vagal Control: Resting, Reactivity, and Recovery

Resting cardiac vagal control has been the focus of most literature considering cardiac vagal control (Thayer et al., 2009), and represents the basis for the main prediction of the neurovisceral integration model, as a higher resting cardiac vagal control (a fuller tank as measured during a resting moment) is associated to positive output at the level of emotion, executive functioning, and health (Thayer et al., 2009). However we argue here that it is important to consider what we refer to as the three Rs of cardiac vagal control: resting, reactivity, and recovery; which all represent different levels of adaptability that are important for human behavior (Laborde et al., 2017b).

Resting refers to a baseline vagal level that is usually measured while people are sitting, with a standard duration of 5 min as recommended by the Task Force (Malik, 1996); however, in specific cases the baseline could also be measured supine or while standing, according to the objectives of the research (Laborde et al., 2017b).

Reactivity represents the change between baseline and a specific event, like completing a task, for example cognitive, emotional, or physical. Reactivity to an event or stress is crucial regarding adaptability and both lower and higher vagal withdrawal can be facilitative when facing demands (Beauchaine et al., 2007). Even if resting cardiac vagal control has been the most investigated parameter together with self-regulation, it happens that certain phenomena reveal themselves only when considering reactivity. For example, a study found that children self-regulation behavior problems were not related to resting cardiac vagal control but only to its reactivity (Calkins et al., 2007). In this case, having considered only resting cardiac vagal control would not have allowed displaying the links between self-regulation and cardiac vagal control. Therefore considering the change in the tank from resting to event is important to understand self-regulation.

Recovery is usually seen as a process of restoration to a former or improved condition. In our case, we would refer to the change between event and a time point after the event (i.e., post-event) where measurement would be taken in similar condition to that of the baseline. Similar to reactivity, recovery plays a crucial role regarding the adaptability of the organism, to face an event and then return to resting level (Stanley et al., 2013). Vagal recovery has also been termed “vagal rebound” in the literature (Nederend et al., 2016). Following our metaphor, recovery deals with whether individuals fill their tank quickly enough to face another stressor, in order to have enough ability to self-regulate and react effectively.

**FIGURE 3:**
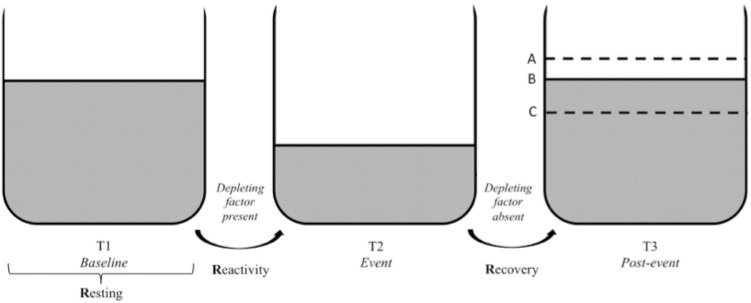
Vagal tank and the 3 Rs of cardiac vagal control: effect of a depleting factor. Illustration of the vagal tank and the three Rs (resting, reactivity, and recovery) with a factor depleting cardiac vagal control. In regards to the post-event: A – displays a situation where the level of carrdiac vagal control during the post-event is higher than the initial level at baseline, B – displays a situation where the level of cardiac vagal control at post-event is similar to the initial level at baseline, and C – displays a situation where the level of cardiac vagal control at post-event did not recover to the point of its initial baseline level.

**FIGURE 4:**
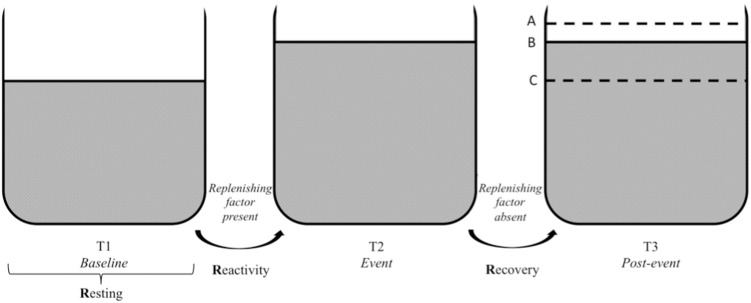
Vagal tank and the 3 Rs of cardiac vagal control: effect of a replenishing factor. Illustration of the vagal tank and the three Rs (resting, reactivity, and recovery) with a factor replenishing cardiac vagal control. In regards to the post-event: A – displays a situation where the level of cardiac vagal control during the post-event is higher than the level during the baseline, B – displays a situation where the level of cardiac vagal control at post-event is similar to the event level, and C – displays a situation where the level of cardiac vagal control at post-event returned to baseline level.

The three Rs of cardiac vagal control fit well the metaphor of the vagal tank, as it is depicted in **Figures 3, 4**. Each of the three levels of adaptability plays a role on self-regulation when individuals face demands. Indeed, the three Rs (i.e., resting, reactivity, and recovery) illustrate the constant role of cardiac vagal control to index self-regulatory behavior before, during, and after the demands faced by the individual. This further demonstrates the overarching role of cardiac vagal control regarding self-regulation processes, which favorably argues its ability to underpin the vagal tank theory.

## Vagal Tank Theory: Research Questions, Predictions, Research Designs, and Unifying Framework

The vagal tank theory, building on previous theoretical accounts regarding self-regulation from a neurophysiological perspective, where cardiac vagal control has been mainly studied (Thayer et al., 2009), and self-regulation from a cognitive and social psychology perspective, which has been looking for a physiological resource (Baumeister et al., 2007; Hagger et al., 2010; Kotabe and Hofmann, 2015), aims to extend our understanding of self-regulation.

In comparison to previous theoretical accounts from neurophysiology on cardiac vagal control, the neurovisceral integration theory (Thayer et al., 2009) and the polyvagal perspective (Porges, 2007b), the main added value of the vagal tank theory is the systematic consideration of the three Rs (i.e., resting, reactivity, recovery) which enables to understand the complexity of behavior, and which leads to new research questions, predictions, and research designs.

In comparison to previous theoretical accounts from a cognitive and social psychology perspective, it will enable the systematic test of a physiological indicator reflecting how efficiently resources can be mobilized and used in self-regulation experiments with a clear theoretical background. From there, it can complement the purely motivational accounts on self-control, which discarded so far the existence of a resource (Kurzban et al., 2013; Inzlicht et al., 2014, 2015), to reach an integrated view of self-regulation combining motivation and cardiac vagal control. Interestingly, previous research already pointed out such links, stating that the influence of cardiac vagal control on development was best understood when integrating motivation (Beauchaine, 2001; Beauchaine et al., 2007). Finally, the vagal tank theory will help to advance self-regulation further from a cognitive and social psychology perspective, allowing to understand factors depleting and replenishing cardiac vagal control, integrating them to traditional cognitive and social psychology research designs.

To sum up at the theoretical level, the vagal tank theory posits that self-regulation considered from a purely neurophysiological perspective on the one hand, and from a cognitive and social perspective on the other hand, can be indexed, at least partially, on the same physiological component, cardiac vagal control. This does not exclude that the links between cardiac vagal control and self-regulation cannot be influenced by some moderators, like motivational processes (Kurzban et al., 2013; Inzlicht et al., 2014, 2015), and this interaction needs to be investigated further.

### Predictions Based on the Three Rs

Vagal tank theory is making specific predictions regarding the three Rs (i.e., resting, reactivity, and recovery), building on theoretical insights from neurophysiology, cognitive, and social psychology.

#### Predictions: Resting Cardiac Vagal Control

In line with the neurovisceral integration model (Thayer et al., 2009) and the polyvagal theory (Porges, 2007b), we predict that a higher cardiac vagal control will be linked to higher executive performance, to better stress management and emotional regulation, to a better social functioning, and to a better overall health (Porges, 2007b; Thayer et al., 2009). Unless specified otherwise, those different domains are grouped under the umbrella term self-regulation in the next hypotheses. This matches the resource view according to Baumeister et al. (2007), and the control capacity according to the integrative theory on self-control (Kotabe and Hofmann, 2015).

Some limitations should, however, be noted to this general prediction, based on evidence from neurophysiology, medicine, and cognitive and social psychology. Regarding neurophysiology and executive functioning, this general hypothesis might not extend to all executive functions, but may be specific to executive functions involving inhibition and working memory components (Kimhy et al., 2013; Jennings et al., 2015). Regarding well-being, cardiac vagal control showed a quadratic relationship with multiple measures of well-being, illustrating the fact that some biological processes may cease being adaptive when reaching extreme levels (Kogan et al., 2013). Regarding health, excessive cardiac vagal control may have potentially deleterious consequences including syncope, pulmonary airway constriction, and increased gastric secretion (Chapleau and Sabharwal, 2011). In addition, abnormal heart rate patterns in the elderly that increase cardiac vagal control indices were found to be linked to increased mortality (Stein et al., 2005); and elevated cardiac vagal control has also been observed in individuals with eating disorders (Peschel et al., 2016), potentially due to a decreased resting metabolic rate originating from limited calorie intake. Moreover, motivation may play a moderator role regarding cardiac vagal control, as depicted by social psychology (Baumeister et al., 2007; Hagger et al., 2010), the motivational accounts (Kurzban et al., 2013; Inzlicht et al., 2014, 2015) and by the integrative theory on self-control (Kotabe and Hofmann, 2015). Hence, the exact way motivation interacts together with the vagal tank needs to be clarified. This moderator role of motivation on self-regulation will have to be considered as well at the other levels of cardiac vagal control analysis, namely reactivity and recovery.

To sum up, further research endeavors should explore to which extent the overall hypothesis regarding resting cardiac vagal control and self-regulation, which we could coin “the higher the better” holds true, and try to delineate its limitations and potential moderators on both a theoretical and physiological point of view. In particular, there is a need to narrow down the general hypothesis to more clearly capture any shared regulation mechanisms, taking in particular into account the nature of the cognitive functions investigated, the age and health condition of the individuals, as well as the existence of potential moderators such as motivation.

#### Predictions: Cardiac Vagal Control Reactivity

Regarding reactivity, both an increase and a decrease can be seen as adaptive according to the demands of the situation (Beauchaine et al., 2007; Porges, 2007b; Thayer et al., 2009). Previous theoretical approaches, such as the neurovisceral integration model (Thayer et al., 2009) and the polyvagal theory (Porges, 2007b) acknowledged the role of cardiac vagal control reactivity in adaptation. The polyvagal theory (Porges, 2007b) states that the successful adaptation of behavior is dependent on systematic reliable withdrawal and reengagement of the vagal brake as a mechanism to rapidly regulate metabolic output in response to environmental demands, so as to match for example the classical fight or flight response (Porges, 1995a; Porges et al., 1996). Overall, it assumes that cardiac vagal control is withdrawn in response to environmental demands which include metabolically demanding states such as exercise, stress, attention, and information processing (Porges, 1995a). Cardiac vagal control withdrawal is expected to be smaller or larger according to the metabolic demands: for example sustained attention to maintain social behavior will be accompanied by a smaller cardiac vagal control decrease, while the fight or flight response will provoke a near complete vagal withdrawal (Beauchaine et al., 2007; Porges, 2007a,b). The large cardiac vagal control withdrawal in this situation facilitates large increases in cardiac output by the sympathetic nervous system, which is no longer being opposed by inhibitory influences. This withdrawal serves as a quick adaptation of the body to meet the demands of the situation, for example as a response to physical activity (Fu and Levine, 2013; Fisher, 2014) or to position change (e.g., orthostatic response, Cavalcante Neto et al., 2018). If the polyvagal theory is helpful to delineate the first outlines of our predictions regarding cardiac vagal control reactivity in terms of metabolic demands, it is somehow limited with its focus on social functioning, its non-specificity regarding cognitive functions, and the fact it does not envisage a cardiac vagal control increase in reaction to the event. In order to precise the predictions of the vagal tank theory regarding cardiac vagal control reactivity, we will then combine the polyvagal theory with the neurovisceral integration model (Thayer et al., 2009, 2012).

If the neurovisceral integration model (Thayer et al., 2009, 2012) did not refer to metabolic demands to understand cardiac vagal control reactivity, it does offer precious insights in terms of executive performance, and emotion regulation relying heavily on top-down functioning. In this case, a smaller cardiac vagal control withdrawal or even an increase in cardiac vagal control during the event would reflect a better self-regulation. Seen like this, Thayer et al. (2012) mentions that both resting cardiac vagal control and its phasic aspect as reactivity could be considered as indicators of the integrity of the resources on which self-regulation is based. Specifically, a cardiac vagal control increase can be expected during the successful regulation of emotion during emotion regulation tasks, which has been showed empirically for example in Park et al. (2014). Moreover, empirical evidence also showed that a smaller decrease in cardiac vagal control leads to better executive performance under pressure, in comparison to a larger cardiac vagal control withdrawal (e.g., Laborde and Raab, 2013; Laborde et al., 2014). On the contrary, for non-executive tasks (i.e., dart throwing task under high pressure), a larger cardiac vagal withdrawal was seen to benefit performance, suggesting that the adaptive character of cardiac vagal reactivity is task and situation dependent (Mosley et al., 2017).

A nice complement regarding reactivity comes from social psychology with the strength model of self-control (Baumeister et al., 1998) and from cognitive psychology with the integrative theory on self-control (Kotabe and Hofmann, 2015). This approach would assume a resource-based functioning for self-regulation, meaning that a smaller decrease (or an increase) would mean more resource available to meet the self-regulatory demands of the task. This is nicely illustrated by Segerstrom and Nes (2007) who mention that in contrast to situations where there is urgent need for the organism to redirect energy to the heart and large muscles in case of fight or flight, self-regulation can also require mental effort, seen here in terms of executive functions, and is often a matter of not acting. Therefore in these cases it may be useful to engage the vagal brake to reduce energy demands in the periphery, and make instead resources available for the metabolic costs of mental effort based on top-down prefrontal functioning, in order to promote calm reflection (Porges, 2001; Fairclough and Houston, 2004; Segerstrom and Nes, 2007).

To summarize, based on theoretical considerations from the neurovisceral integration model (Thayer et al., 2009, 2012) and the polyvagal theory (Porges, 1995b, 2007b) on the one hand, and based on the resource approach from cognitive and social psychology on the other hand (Baumeister et al., 2007; Kotabe and Hofmann, 2015), we have to distinguish between situations requiring low level of activity from situations requiring high level of activity, and according to how much top-down executive processing is needed to face the situation. In case a higher level of activity is required by the situation, when exposed to direct stress, and when metabolic demands are important, we would hypothesize that a higher vagal withdrawal is associated to a better self-regulation performance (Porges, 1995b, 2007b; Thayer et al., 2012; Park et al., 2014). However, when the task requires a low level of activity and highly relies on executive functioning and top-down control, we suggest that a smaller vagal withdrawal is seen as adaptive (Porges, 1995b, 2007b; Thayer et al., 2012; Park et al., 2014).

From this prediction regarding reactivity, we understand that we should not consider cardiac vagal control as a resource itself (i.e., depletion always having negative consequences), because for example a stronger cardiac vagal control withdrawal would be seen as more adaptive in specific situations requiring higher metabolic costs, which would deviate from the vision as a resource from the cognitive and social psychology perspective (Baumeister et al., 2007; Kotabe and Hofmann, 2015). Rather, considering here reactivity, we argue for the patterns of change in cardiac vagal control to reflect the effectiveness of the self-regulation mechanisms of the organism.

#### Predictions: Cardiac Vagal Control Recovery

None of the theoretical approaches on cardiac vagal control, neither the neurovisceral integration model (Thayer et al., 2009) nor the polyvagal theory (Porges, 2007b), clearly make predictions regarding cardiac vagal control recovery, while this aspect is central regarding the adaptation of the organism (Stanley et al., 2013), and in building resting cardiac vagal control on the long-term.

Regarding recovery, we need to distinguish two situations: a cardiac vagal control increase during the event, or a cardiac vagal control decrease during the event. After a vagal withdrawal during the event, a return to initial resting levels or higher at the post-event time point is seen as adaptive (Stanley et al., 2013). This is because the adaptive individual has experienced a demand and as a result cardiac vagal control decreased but has the necessary means to return to baseline levels in order to face a new demand or be fully recovered. More specifically, the faster cardiac vagal control level comes back to initial level, the better the self-regulation (Stanley et al., 2013). Following this view, the resource account from the cognitive and social psychology perspective (Baumeister et al., 2007; Kotabe and Hofmann, 2015) would assume that self-regulation effectiveness can be restored once the resource is replenished, therefore the faster the tank returns to baseline levels the better. Further, a link between performance and subsequent cardiac vagal recovery may be established: for example, a recent study showed that cardiac vagal control recovery was directly linked to performance outcomes (i.e., prone rifle shooting), in that those who had superior performance had a faster cardiac vagal control recovery than those who performed poorly (Mosley et al., 2018). This suggests those who had a poor performance were not able to recover effectively from the event. This would imply to control whenever possible for task performance in order to better understand cardiac vagal control recovery. Finally, on the one hand, we know that after vagal withdrawal due to high physiological demands, vagal recovery occurs faster in individuals with greater aerobic fitness (Stanley et al., 2013), while on the other hand, delayed cardiac vagal control recovery reflects self-regulation dysregulation (Berna et al., 2014). To sum up, we predict that the faster cardiac vagal control level comes back to initial level, the better the self-regulation.

If there is an increase of cardiac vagal control during the event, while the demand is being faced, we would assume that cardiac vagal control staying the longer at this level would be the most suitable regarding adaptation for recovery and post-event, because on the long-run it may help build a higher resting cardiac vagal control (based on Thayer et al., 2009; Stanley et al., 2013). Therefore, in case cardiac vagal control increased during the event, we would assume that the longer cardiac vagal control stays above initial resting level, the better the self-regulation.

Similar to resting cardiac vagal control, the links between the general hypothesis and specific outputs need to be tested. For example, it was found that a faster cardiac vagal control recovery after a cognitive challenge was associated with reduced deficits in executive function among older individuals, but not among younger individuals (Crowley et al., 2016).

#### Predictions: Interaction Patterns Between the Three Rs

Importantly, taking into account systematically the 3 Rs will also enable to investigate the interaction pattern between them. When talking about cardiac vagal control adaptive properties concerning cardiac vagal control reactivity and recovery, we refer to the specific predictions related to cardiac vagal control reactivity and recovery stated in the previous sections.

The first pattern is a link between resting cardiac vagal control and cardiac vagal control reactivity and recovery. According to the neurovisceral integration model (Thayer et al., 2009, 2012), as a higher resting cardiac vagal control should promote a more adaptive functioning, we would expect that it triggers as well a more adaptive cardiac vagal control reactivity and recovery. For reactivity for example, considering a selective attentional task with varying levels of load, a lower resting cardiac vagal control was associated to cardiac vagal control suppression, suggesting an autonomic stress response, under both low and high load. While a high resting cardiac vagal control was associated with cardiac vagal control enhancement under low load, suggesting greater self-regulatory effort, and an absence of phasic cardiac vagal control suppression under high load (Park et al., 2014). On the contrary, the combination of a low resting cardiac vagal control and a higher cardiac vagal control decrease may be maladaptive, this is why cardiac vagal control over-reactivity is used as marker of emotion dysregulation (Beauchaine, 2015). Regarding recovery, we would expect similarly that a higher resting cardiac vagal control is linked to a more adaptive recovery. For example, a lower resting cardiac vagal control was associated to a delayed cardiac vagal control recovery after a stressful event (Weber et al., 2010), while people with higher fitness level, and hence with higher resting cardiac vagal control, display a faster cardiac vagal control recovery (Stanley et al., 2013).

The second pattern is a link between reactivity and recovery, and we would assume that a more adaptive cardiac vagal control reactivity associated to a more adaptive cardiac vagal control recovery predicts a better self-regulation. For example, a pattern of cardiac vagal control decrease in response to angry event recall, and subsequent cardiac vagal control increase (i.e., both cardiac vagal control reactivity and recovery can be considered as adaptive in this case) were related to better anger and sadness regulation and more prosocial behavior (Cui et al., 2015).

Finally, we highlight that interaction patterns can emerge and reveal relationships that were not evidenced by considering the three Rs in isolation. For example, in one study it was found that resting cardiac vagal control and cardiac vagal control reactivity were independently unrelated to depression, but their interaction predicted latent depression levels (Yaroslavsky et al., 2013).

To sum up, investigating systematically the three Rs enables to make predictions regarding interactions and specific cardiac vagal control response patterns, which potentially would have not emerged if each of the time point would have been investigated independently. Hence, this systematic investigation of the three Rs may help to shed light on findings that would otherwise not make sense. The role of resting cardiac vagal control on cardiac vagal control reactivity and recovery also highlights how important is resting cardiac vagal control. This is described by Grossman and Taylor (2007) as they state that resting cardiac vagal control reflects a “functional energy reserve capacity from which the organism can draw during more active states” (p. 279), which would also fit the resource metaphor from the strength model on self-control (Baumeister et al., 2007), and the control capacity view of the integrative self-control theory (Kotabe and Hofmann, 2015). A higher initial level of cardiac vagal control, supposed to depict the effectiveness of self-regulatory mechanisms, would therefore underpin more adaptive reactivity and recovery phases. An overview of all predictions of the vagal tank theory can be seen in **Table 1**.

**Table 1:** Summary of the predictions of the vagal tank theory, based on the three Rs (Resting, Reactivity, Recovery).

	Three Rs	Specification	Prediction
Adaptation level	Resting		“The higher the better”: Better self-regulation with higher resting cardiac vagal control
	Reactivity	Situation requires a low level of physical activity and relies mainly on executive functioning	Better self-regulation with smaller cardiac vagal control decrease (or in some cases cardiac vagal control increase)
		Situation requires a high level of physical activity	Better self-regulation with larger cardiac vagal control decrease
	Recovery	Cardiac vagal control decreased during the event	The faster cardiac vagal control level comes back to initial level, the better the self-regulation
		Cardiac vagal control increased during the event	The longer cardiac vagal control stays above initial resting level, the better the self-regulation
Interaction patterns	Resting-Reactivity		A higher resting cardiac vagal control predicts a better self-regulation during reactivity
	Resting-Recovery		A higher resting cardiac vagal control predicts a better self-regulation during recovery
	Reactivity-Recovery		A more adaptive cardiac vagal control reactivity associated to a more adaptive recovery predicts a better self-regulation than when only either the reactivity or the recovery is adaptive

*As detailed in the manuscript, self-regulation is used here as an umbrella term and encompasses executive functioning, stress management, emotional regulation, social functioning, and overall health. In addition, for each aspect of the three Rs, some limitations have to be taken into account concerning the predictions: First, regarding executive functioning, the predictions may be limited to specific executive functions involving primarily inhibition and working memory. Second, regarding health, in specific cases an excessive cardiac vagal control may be associated to deleterious consequences such as syncope, pulmonary airway constriction, and increased gastric secretion. Finally, motivation may play a moderator role regarding each prediction.*

### Vagal Tank Theory: Implications in Terms of Research Designs

At the level of research design, vagal tank theory is aimed to test systematically the three main aspects of cardiac vagal control regarding adaptation: resting, reactivity, and recovery. This means that all research designs willing to test thoroughly all adaptive aspects of cardiac vagal control and aiming to test the predictions of the vagal tank theory need to integrate measurements of cardiac vagal control at rest, during the event, and during a recovery period. Those different measurements should be equivalent in time and realized in the same body position, in order to offer a meaningful comparison, following when possible the 5 min standard guideline of the Task Force (Malik, 1996). If the task is shorter than 5 min, resting and recovery time can be adjusted in consequence; if the task is longer than 5 min, then resting and recovery can follow the 5-min rule, and then the task could be cut during the analysis in meaningful segments matching resting and recovery time. Further methodological considerations regarding cardiac vagal control assessment and research designs including cardiac vagal control can be found in several summary works (Quintana and Heathers, 2014; Quintana, 2016; Quintana et al., 2016; Fatisson et al., 2016; Laborde et al., 2017b).

## Conclusion

The aim of this paper was to set the stage for the vagal tank theory, enabling a shift in self-regulation understanding, combining approaches from neurophysiology on the one hand, and from cognitive and social psychology on the other hand, enabling to advance previous work on self-regulation at the theoretical, methodological, and applied levels. Obviously, we are not arguing that cardiac vagal control alone determines behavior, however we made here the case through the vagal tank theory that cardiac vagal control has an overreaching influence on several key self-regulatory aspects of behavior.

The vagal tank theory advances previous neurophysiological theories, namely the neurovisceral integration model (Thayer et al., 2009) and the polyvagal theory (Porges, 2007b), building on them and offering a systematic investigation of the three Rs of cardiac vagal control, namely resting, reactivity, and recovery. Each of these levels of analysis is associated to specific predictions, and a case was made for the investigation of their interaction pattern. Moreover, it advances cognitive and social psychology approaches (Baumeister et al., 2007; Kurzban et al., 2013; Inzlicht et al., 2014, 2015; Kotabe and Hofmann, 2015), where the debate around a potential physiological resource has surrounded the field since its early beginnings, proposing a physiological indicator indexing the mechanisms underlying self-regulation. As we presented above, we do not see cardiac vagal control as a resource itself. If the resource metaphor would fit resting cardiac vagal control and cardiac vagal control recovery, it is not strictly the case for cardiac vagal control reactivity, given sometimes a higher decrease is more adaptive when the metabolic demands of the situation require it. Another point is that it is very likely based on its physiological origins that cardiac vagal control is actually not a resource that gets used, but reflects more the integrity and adaptability of the general self-regulation mechanisms of the organism (Porges, 2007b; Thayer et al., 2009, 2012). Still, the vagal tank metaphor and its predictions regarding the way it gets depleted and replenished helps to understand further the self-regulation mechanisms underlying human behavior. The predictions of the vagal tank theory certainly need to be empirically tested combining the specific methodologies of the different theoretical approaches presented above. This would allow for delineation of the predictions, in terms of limitations and potential moderators, which would ultimately enable a more comprehensive understanding of self-regulation.

At the level of research designs, the consequences of vagal tank theory are that researchers need to include the evaluation of the three Rs (i.e., resting, reactivity, recovery) within their experiment in order to reach a better comprehension of the phenomena under investigation (Laborde et al., 2017b). Hence, the vagal tank theory has the potential to deeply transform research on self-regulation made from both a neurophysiology perspective on the one hand, and cognitive and social psychology on the other hand, because in the former case the three Rs were very rarely systematically investigated (for an exception, see Berna et al., 2014), while in the latter case cardiac vagal control has been almost never considered so far (for exceptions, see Segerstrom and Nes, 2007; Reynard et al., 2011).

The vagal tank theory also offers stimulating applied perspectives, in many environments, such as medicine, school, work organizations, sports, and everyday life. Having a general index of self-regulation such as cardiac vagal control enables to understand better the self-regulation demands of tasks, to build interventions, and assess their effectiveness with an objective physiological marker. Moreover, the availability of small and light devices to assess cardiac vagal control, and the availability of smart phones apps able to assess it reliably (Flatt and Esco, 2013), makes it a very powerful tool to provide a constant insight on the ability of individuals to self-regulate. This ambulatory monitoring of cardiac vagal control enables an easy transfer from the lab to the field, which can become very insightful when coupled to other methodologies such as diaries or questionnaires (Segerstrom and Nes, 2007).

Cardiac vagal control allows as well to test self-regulation mechanisms in non-conscious patients, or in conditions where complex experimental conditions and data collection are hard to realize (Riganello et al., 2012). In addition, cardiac vagal control has been shown to be associated to self-regulation mechanisms across the lifespan, for example already in fetuses cardiac vagal control is a marker of self-regulation (Groome et al., 1999), and cardiac vagal control has a prognostic value in the elderly (Nicolini et al., 2012). However, the fact that cardiac vagal control assessment is relatively easy to realize, non-invasive, and cost-effective, should not cover the fact that strict measurement rules are to be followed if one wants to get meaningful information out of it, for example in terms of controlling for movement, respiration, etc., and that interpretation of results requires to be done with caution (Malik, 1996; Quintana and Heathers, 2014; Shaffer et al., 2014; Laborde et al., 2017b). Carefully scrutinizing methodological differences that can account for disparate findings will also help develop further the vagal tank theory, and potentially help the development of certain testing conditions to become standard, in order to enhance comparability of study findings. Further, the vagal tank theory focuses on the contribution of the vagus nerve to cardiac functioning, however the vagus nerve innervates many other organs of the parasympathetic nervous system than the heart, such as the gut (Brodal, 2010), which also may play a role in self-regulation (Sarkar et al., 2016; Allen et al., 2017). Therefore, future research should investigate how to extend the vagal tank theory considering for example the brain-gut-microbiome axis, which has been found to be linked to many self-regulatory phenomena at the psychological and physiological level (Sarkar et al., 2016; Allen et al., 2017).

To conclude, the vagal tank theory has a strong heuristic predictive value, to help people understand how the vagal tank sustains their self-regulatory efforts, and how this tank gets depleted and replenished when considering the changes in cardiac vagal control. Some questions will naturally arise, such as knowing the factors influencing the three three Rs of cardiac vagal control. For example, what are the factors helping to build a higher resting cardiac vagal control over time? What are the factors influencing cardiac vagal control reactivity and recovery? Answering those questions may contribute on a theoretical level to advance the vagal tank theory, while at the applied level, they may have a strong impact on our individual lives and our functioning in society, triggering an increased awareness about the factors depleting and replenishing the vagal tank. For example, it would be important to know what to do when the vagal tank is depleted. A fix of self-regulation failure could be realized for example with a mindfulness training (Brewer et al., 2009; Libby et al., 2012) or with slow paced breathing (Wells et al., 2012; Laborde et al., 2017a), which would contribute to increasing cardiac vagal control. We would like to conclude with the words of Kurt Lewin: “There is nothing as practical as a good theory” (Lewin, 1952, p. 346), and we hope that the heuristic visualization offered by the vagal tank theory may help people to become aware on how to take action on their self-regulation abilities.

## Author Contributions

SL prepared the manuscript. EM and AM provided critical feedback to improve it.

## Conflict of Interest Statement

The authors declare that the research was conducted in the absence of any commercial or financial relationships that could be construed as a potential conflict of interest.
